# Supplementary material for: Anserine, a Histidine-Containing Dipeptide, Suppresses Pressure Overload-Induced Systolic Dysfunction by Inhibiting Histone Acetyltransferase Activity of p300 in Mice
Source: Int J Mol Sci. 2024 Feb 16;25(4):2344. doi: 10.3390/ijms25042344 (PMC10889817; doi:10.3390/ijms25042344)
Supplement: Supplementary file 1 [file ijms-25-02344-s001.zip › ijms-2845210-supplementary.pdf]

Supplemental Figure S1

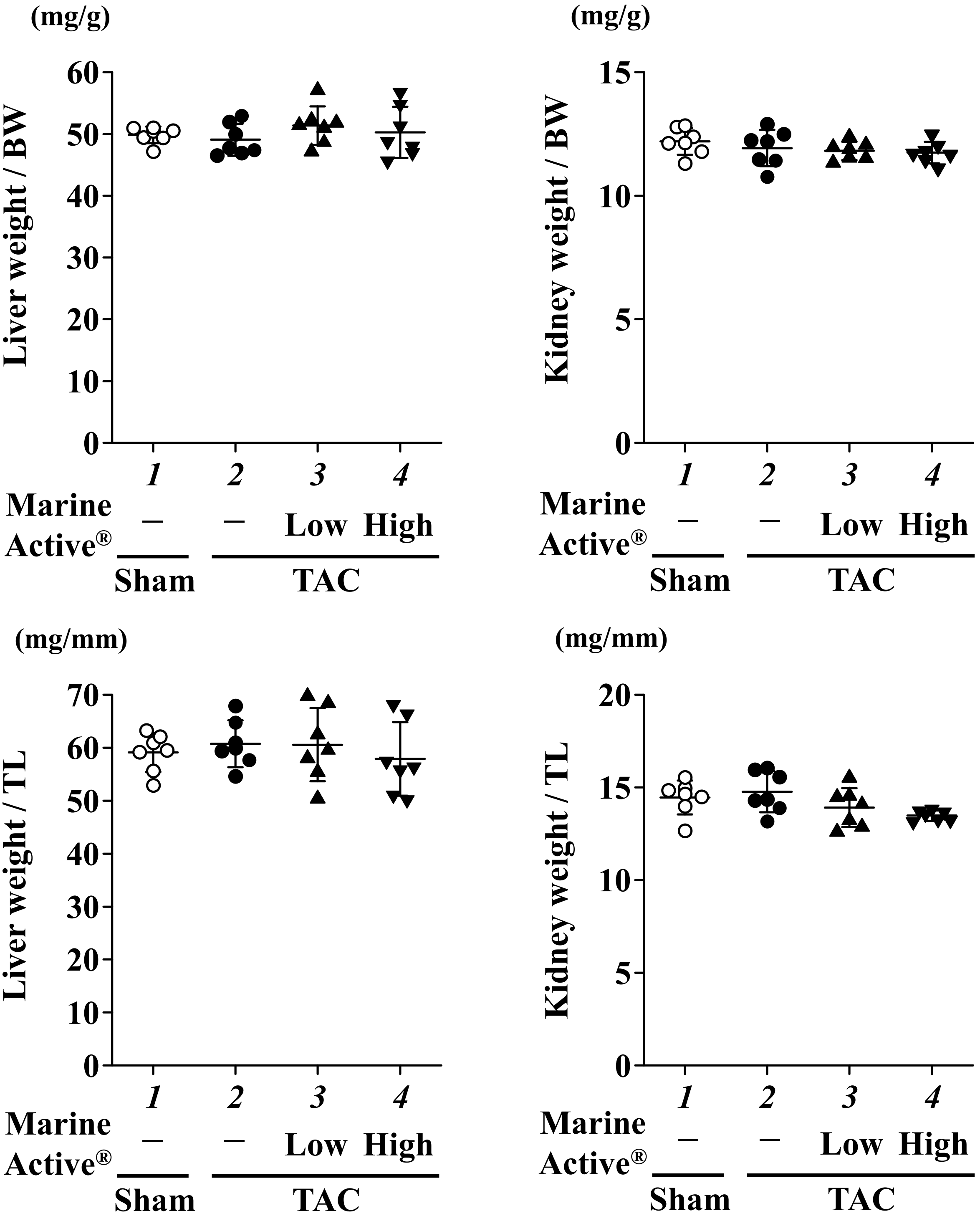

Supplemental Figure S1 Liver and kidney weights to BW or TL ratios at 8 weeks after TAC operation

(**A**-**D**) The ratio of liver weight-to-BW (**A**), kidney weight-to-BW (**B**), liver weight-to-TL (**C**), and kidney weight-to-TL (**D**) are presented as dot plots. Data are presented as the means ± SD (n=7).

Supplemental Figure S2

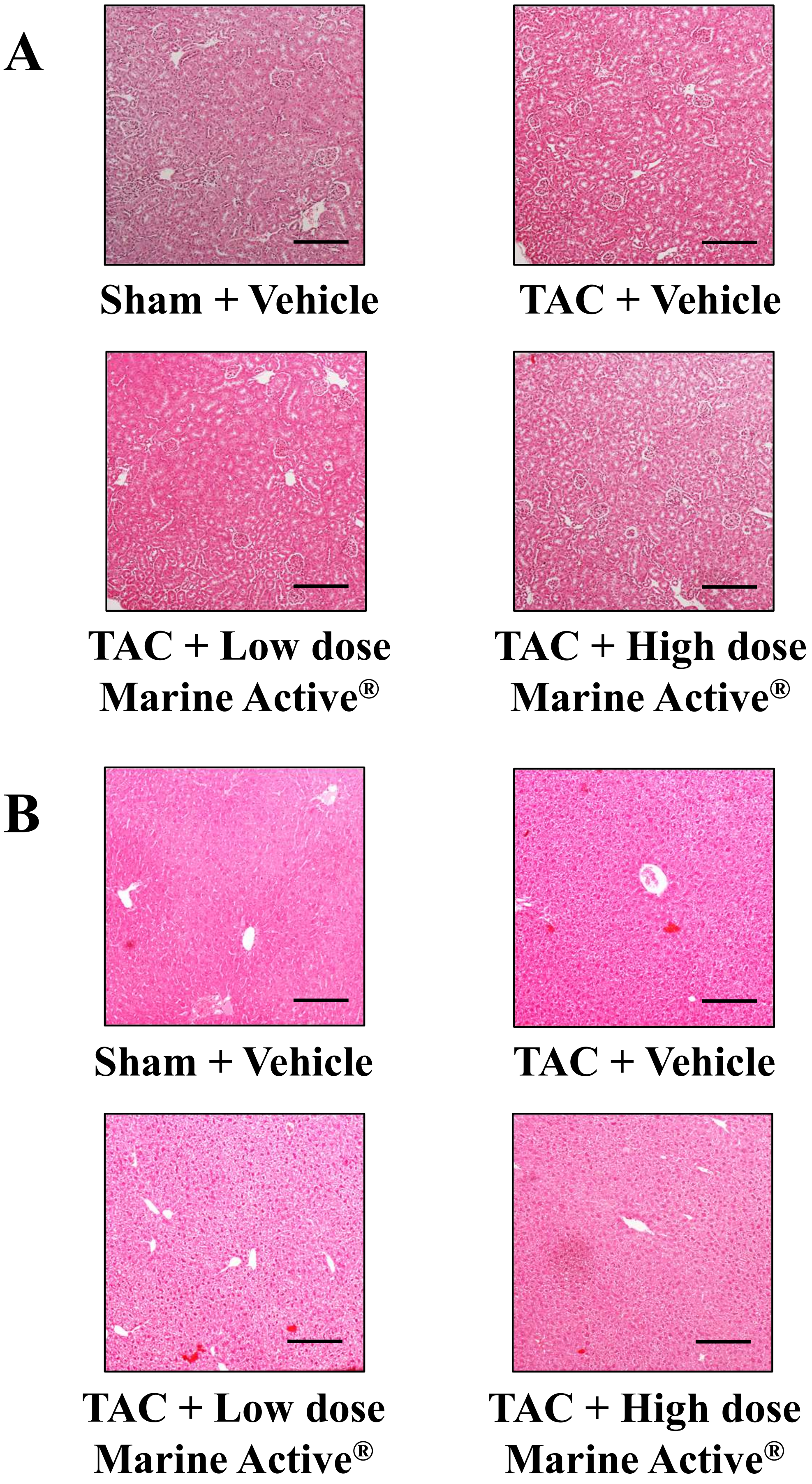

Supplemental Figure S2 Microscopic photographs of liver and kidney tissues.

(**A** and **B**) Representative microscopic images of liver (**A**) and kidney (**B**) with HE staining were visualized by light microscopy. Scale bar: 200 µm.
